# Physiological and Metabolic Responses of Gac Leaf (*Momordica cochinchinensis* (Lour.) Spreng.) to Salinity Stress

**DOI:** 10.3390/plants11192447

**Published:** 2022-09-20

**Authors:** Thitiwan Jumpa, Diane M. Beckles, Patcharin Songsri, Kunlaya Pattanagul, Wattana Pattanagul

**Affiliations:** 1 Department of Biology, Faculty of Science, Khon Kaen University, Khon Kaen 40002, Thailand; 2 Department of Plant Sciences, University of California, Davis, CA 95615, USA; 3 Department of Plant Sciences and Agricultural Resources, Faculty of Agriculture, Khon Kaen University, Khon Kaen 40002, Thailand; 4 Department of Statistics, Faculty of Science, Khon Kaen University, Khon Kaen 40002, Thailand

**Keywords:** gac, salinity stress, physiological traits, metabolic profile

## Abstract

Gac is a carotenoid-rich, healthful tropical fruit; however, its productivity is limited by soil salinity, a growing environmental stress. This study aimed to evaluate the effects of salinity stress on key physiological traits and metabolites in 30-day-old gac seedling leaves, treated with 0, 25-, 50-, 100-, and 150-mM sodium chloride (NaCl) for four weeks to identify potential alarm, acclimatory, and exhaustion responses. Electrolyte leakage increased with increasing NaCl concentrations (*p* < 0.05) indicating loss of membrane permeability and conditions that lead to reactive oxygen species production. At 25 and 50 mM NaCl, superoxide dismutase (SOD) activity, starch content, and total soluble sugar increased. Chlorophyll *a*, and total chlorophyll increased at 25 mM NaCl but decreased at higher NaCl concentrations indicating salinity-induced thylakoid membrane degradation and chlorophyllase activity. Catalase (CAT) activity decreased (*p* < 0.05) at all NaCl treatments, while ascorbate peroxidase (APX) and guaiacol peroxidase (GPX) activities were highest at 150 mM NaCl. GC-MS-metabolite profiling showed that 150 mM NaCl induced the largest changes in metabolites and was thus distinct. Thirteen pathways and 7.73% of metabolites differed between the control and all the salt-treated seedlings. Salinity decreased TCA cycle intermediates, and there were less sugars for growth but more for osmoprotection, with the latter augmented by increased amino acids. Although 150 mM NaCl level decreased SOD activity, the APX and GPX enzymes were still active, and some carbohydrates and metabolites also accumulated to promote salinity resistance via multiple mechanisms.

## 1. Introduction

Gac (*Momordica cochinchinensis* (Lour.) Spreng.), also known as a sweet gourd, belongs to the Cucurbitaceae and is popular in South and South-East Asia because of its intense red color and high carotenoid content [1], which is 10- and 70-fold higher than in carrots and tomatoes, respectively. While *β*- carotenes, and lycopene are abundant in gac, zeaxanthin and *β*-cryptoxanthin also accumulate to high levels [2]. Gac fruit is therefore an effective and new and valuable source of carotenoids which may contribute significant benefits to human health [3]. Daily consumption of “gac” enhanced plasma levels of retinol, α- and β- carotenes, and, lycopene in children [4], and gac seed membranes promote healthy vision [1]. Therefore, the cultivation of the plant should be encouraged [1,3,4,5,6,7,8,9,10,11].

Soil salinity is a major factor limiting crop productivity worldwide [12,13,14,15]. Salt accumulation in soils develops from unbalanced irrigation, inferior drainage, incorrect application of fertilizer, and overcultivation [16]. Plants growing in saline soils develop complex but highly coordinated morphological, physiological, and metabolic processes to cope with stress [13,14,15,17,18,19,20]. High salt concentrations induce loss of intracellular water, because of the lower soil water potential, hyperionic stress due to the increased levels of ions such as Na^+^ and Cl^−^, and nutrient imbalances caused by the inhibition of essential nutrient uptake, and/or shoot transport. Salinity stress also leads to the overproduction and overaccumulation of reactive oxygen species (ROS) in plant cells, which results in oxidative damage to cellular components such as membranes, proteins, and DNA [21,22,23,24,25]. Plants, however, can modulate ROS accumulation during salinity stress via non-enzymatic and enzymatic antioxidants such as guaiacol peroxidase, (GPX), superoxide dismutase, (SOD), catalase, (CAT), ascorbic peroxidase (APX) [26,27,28,29,30,31,32,33].

The ability of plants to deal with salt stress is an essential determinant of crop distribution and productivity in many areas; thus, understanding the mechanisms that provide tolerance is essential for ensuring productivity. For example, reconfiguration of plant metabolic pathways is an important response [34,35], and identifying key compounds altered as a result may contribute to the understanding salinity resistance mechanisms in plants [36]. The accumulation of antioxidants, amino acids, and sugars can provide protection against salinity stress [23,37,38,39,40,41,42,43], as demonstrated in a variety of species and tissues [26,44,45,46,47,48,49,50]. However, changes in metabolites will depend on the severity or the length of the stress; the metabolites accumulated may be components of the early stress perception response, the acclimation response, or may be metabolic byproducts after stress exhaustion. A better understanding of the relative abundance and spatiotemporal occurrence of these metabolites could help identify tolerance mechanisms in species of interest [34].

There is limited knowledge of the response of gac plant to soil salinity. Our preliminary data found that 4-month-old gac plants grow normally in soil from 25 to 150 mM NaCl, while 200 mM NaCl induces abnormal symptoms [51]. The high level of natural antioxidants in gac may undergird its apparent tolerance to salinity stress. However, metabolic profiles in this plant have been little studied, especially under salinity conditions. Therefore, it is important to investigate the effect of salinity stress on cellular metabolism, in terms of antioxidant enzymes, plant physiological characteristics together with metabolic profiles. The finding of this study will fill this current gap in knowledge and aid in the understanding of metabolic responses in gac plants under salinity stress, which would benefit breeding program aimed at developing salt-tolerant cultivars.

## 2. Materials and Methods

### 2.1. Plant Materials 

Gac seeds (*Momordica cochinchinensis* (Lour.) Spreng.) cv. Kaenpayorm1, were supplied by the Faculty of Agriculture, Khon Kaen University, Khon Kaen, Thailand. Seeds were soaked in distilled water for 2 days and then germinated in saturated peat moss for 7 days. Approximately 5 kg of natural sandy loam soils were transferred to each plastic pot (8 inches in diameter), and tap water was added to saturate the soil before planting. The gac seedlings were watered with tap water every other day in the greenhouse under natural light, temperature, and humidity (June 2017 at Khon Kaen, Thailand). At 30 days post germination, the salinity treatment was irrigated every other day with either 50 mL of 0, 25, 50, 100, or 150 mM of NaCl. Preliminary studies showed that 200 mM NaCl was lethal, therefore the highest concentration used was 150 mM. There were six individual plants for each salt treatment and leaf tissues were harvested 30 days after the application of the stress. 

### 2.2. Carbohydrate Analysis

Total soluble sugar was extracted from approximately 50 mg gac leaf by 9 mL of 80% (*v*/*v*) ethanol. Total sugar content was analyzed using the phenol sulfuric method [52]. One hundred microliters of the sugar solution were put into a glass tube and mixed with 500 µL 5% (*w*/*v*) phenol, 1000 µL concentrated sulfuric acid, and then left for 10 min. The mixture was vortexed and left for 30 min, and then the absorbance was measured at 490 nm with a spectrophotometer (ThermoScientific™ GENESYS™ 20 Visible Spectrophotometer). A mixture of phenol and sulfuric acid without sugar extract was used as a blank. Total sugar content was determined from a standard curve using a known concentration of glucose as a standard. 

Starch was assayed using the ethanol insoluble fraction. Approximately 400 mL of 2M potassium hydroxide was added, and the mixture was incubated in a 90 °C water bath for an hour, and then cooled to room temperature. To each tube, 600 µL of 10% (*v*/*v*) acetic acid was added. The leaf was added with 1 mL of 280 U amyloglucosidase (Sigma Chemical) which had been dialyzed against 0.1 M Na-acetate buffer (pH 4.5), was used to digest the starch to glucose, then incubated in a 45 °C water bath overnight [52]. Starch content was determined from glucose equivalents released after the enzymatic digestion, using hexokinase, and glucose-6-phosphate dehydrogenase (G6PDH) [53]. Twenty microliters of the starch extract were pipetted into a glass tube and mixed with 1 mL glucose assay mixture: 50 mM Tris-HCl (pH 8.0), 100 mM ATP, 500 mM MgCl_2_, 100 mM NAD, 5 µL hexokinase, and 5 µL G6PDH, then mixed by shaking for 30 min. The absorbance was determined at 360 nm with a spectrophotometer. The starch content was determined from a standard curve using a known concentration of glucose as a standard. 

### 2.3. Enzyme Activities

Leaf tissue (0.2 g) was ground on ice using a mortar and pestle in 4 mL of grinding buffer: 50 mM potassium phosphate buffer (pH 7.8), 0.4 mM EDTA, and 1 mM ascorbic acid. The extracts were filtered through 8 layers of cheesecloth, then transferred to a microfuge, and was centrifuged at 8944 g for 1 min. All steps were performed at 4 °C. A 1 mL aliquot of the supernatant was used as a crude extract for the determination of antioxidant enzymes activity including superoxide dismutase (SOD; EC 1.15.1.1), catalase (CAT; EC 1.11.1.6), guaiacol peroxidase (GPX; EC 1.11.1.7), and ascorbate peroxidase (APX; EC 1.11.1.11) [54]. Protein content was investigated using the Bradford method [55]. 

SOD activity was assayed by measuring its ability to inhibit the photochemical reduction of nitro blue tetrazolium chloride (NBT) [56]. The photo-reduction of NBT was measured at 560 nm. One unit of SOD was defined as the amount of enzyme that produced 50% inhibition of NBT. CAT activity was determined by measuring the rate of disappearance of H_2_O_2_ [57]. The subsequence decomposition of H_2_O_2_ was observed at 240 nm. GPX activity was calculated from the amount of tetraguaiacol formed per minute at 470 nm [58]. For the APX assay, the subsequent decrease in ascorbic acid was observed at 290 nm [58].

### 2.4. Chlorophyll Concentration

Chlorophyll concentration was determined according to the method outlined by Arnon [59]. Leaf samples (20 mg) were extracted in 10 mL of 80% (*v*/*v*) acetone. The absorbance of the extract was determined by spectrophotometer at 645 and 663 nm using 80% (*v*/*v*) acetone as a blank. Chlorophyll was also assayed using the Soil Plant Analysis Development (SPAD)-502 m. Ten independent SPAD measurements were made per plant using several different leaves at the position 2/3 of the distance from the leaf base to the apex [60,61].

### 2.5. Electrolyte Leakage

Electrolyte leakage was measured using an electrical conductivity meter [62]. Leaf discs were placed in a test tube containing 10 mL distilled water. The samples were incubated at room temperature for 24 h. The electrical conductivity of the solution (EC_1_) was then read after incubation. The same samples were then placed in a boiling water bath for 20 min and a second EC reading (EC_2_) was taken after cooling the solution to room temperature. Electrolyte leakage was then calculated as EC_1_/EC_2_ and expressed as a percentage.

### 2.6. Metabolite Profiling

Analytical grade solvents and reagents were purchased from Sigma Aldrich (Australia). Samples were extracted in a pre-chilled solution of methanol (pH 7), chloroform, and deionized water (18.2 MΩ) in proportions of 5:2:2 (*v*/*v*/*v*). Approximately 20 ± 5 mg fresh weight of gac leaves were put into Eppendorf tubes and ground to a fine powder in liquid nitrogen. One mL of pre-chilled extraction solution was added to each tube to prevent thawing of the sample. The samples were vortexed for 10 s and centrifuged for 2 min at 17,530× *g*. Five hundred µL of supernatant was dried in the speed vacuum concentrator (LaboGene Brand) and submitted for derivatization to West Coast Metabolomics Center at the University of California Davis, Genome Center, Davis, CA, USA. 

Gas chromatography-mass spectrometry analysis was performed on a total of 30 samples and multivariate analysis was used to compare the metabolite profiles. The data are acquired using the following chromatographic parameters, with more details to be found in Fiehn et al. [63].

### 2.7. Statistical Analysis and Data Analysis

The experimental design was a completely randomized design (CRD) with six replications for the evaluation of all variables. For physiological data, statistical analysis using one-way ANOVA and all means were separated at the *p* < 0.05 level using Duncan’s multiple range test using the SPSS. A student *t*-test at *p* < 0.05 were analyzed to detect significant changes between the mean of metabolites in different concentrations of NaCl. For multivariate statistical analysis, metabolites data were first normalized to an internal standard. Principal component analysis (PCA), partial least squares discriminant analysis (PLS-DA), and orthogonal partial least squares discriminant analysis (OPLS-DA) were performed using Simca^®^.

Scree plot, one-way ANOVA analysis, box plots, heat map, volcano plots, and pathway impact were performed using MetaboAnalyst Version 5.0 (statistical, functional, and integrative analysis of metabolomics data) is freely available at http://www.metaboanalyst.ca/ (accessed on 15 January 2022) [64,65,66]. The identified metabolites were mapped on the KEGG pathway of the model plant *Arabidopsis thaliana*, which demonstrated the role of different metabolites in various biochemical pathways.

## 3. Results

### 3.1. Electrolyte Leakage 

**Figure 1:**
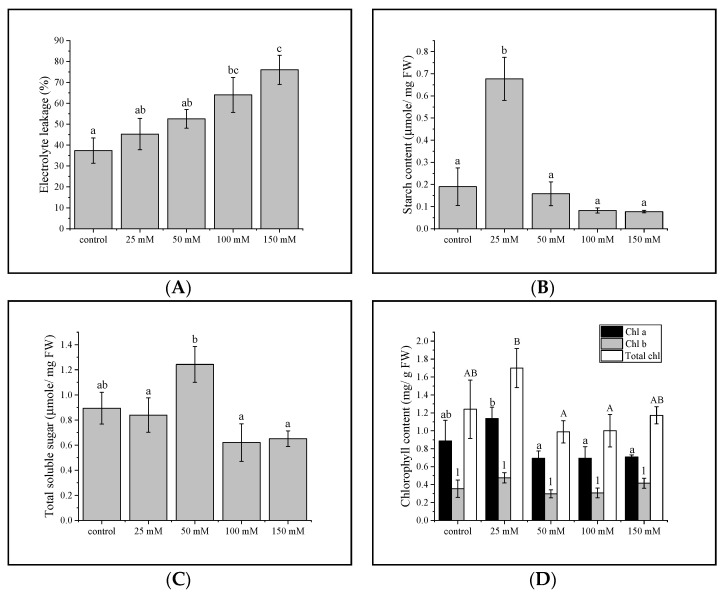
Gac seedlings leaf response to increasing soil salinity. (**A**) Rate of electrolyte leakage, (**B**) starch content, (**C**) total soluble sugar. Different lowercase letters indicate significant differences between treatments (*p* < 0.05). (**D**) Chlorophyll a, b and total chlorophyll in gac leaf at the seedling stage. Values are means ± SE (*n* = 6). Lower case letters, numbers, and capital letters denote significant differences in chlorophyll a, chlorophyll b, and total chlorophyll content, respectively, between treatments (*p* < 0.05).

There was a significant positive relationship between electrolyte leakage in gac seedling leaves and soil salinity (*p <* 0.01; *R*^2^ = 0.3679) (Figure S2). Membrane permeability differed from the control only at 100 mM (*p* < 0.05) and plants treated with 150 mM NaCl were distinct from all others (Figure S1) as well as soil electroconductivity (Figure 1A).

### 3.2. Total Sugar and Starch Content 

Starch content in gac seedling leaves increased when grown under 25 mM NaCl compared to the control plant, while treating with 50 mM NaCl showed similar starch content to the control plant. At 100 and 150 mM NaCl, however, starch content decreased compared to the control plant and 50 mM NaCl but it was not significant (*p* > 0.05) (Figure 1B). Sugar content increased only at 50 mM compared to the control plant. Although 100 and 150 mM NaCl led to reductions in sugar content, they were not significant (*p* > 0.05) (Figure 1C).

### 3.3. Chlorophyll Concentration in Gac Seedling Leaf

Although not significant, the total chlorophyll, chlorophyll *a* content, and SPAD readings of gac seedling leaves showed an increase at 25 mM but decreased (*p* > 0.05) at 50 and 100 mM NaCl compared to the control (Figure 1D). Unlike the chlorophyll readings obtained from the spectrophotometer, SPAD showed an unambiguous decrease at 150 mM NaCl compared to the control (Figure S3), and in contrast to the chlorophyll *a* and total chlorophyll measurements, chlorophyll b, the content was similar at all NaCl concentrations. 

### 3.4. Antioxidant Enzyme Activities 

**Figure 2:**
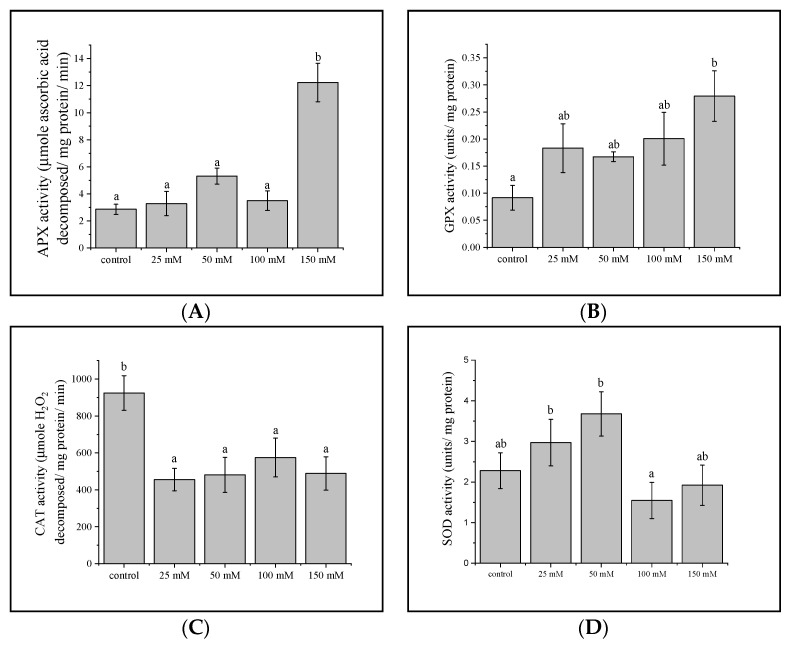
Antioxidant enzyme activity in gac leaves in response to salinity stress. Activity assayed included (**A**) APX, (**B**) GPX, (**C**) CAT, and (**D**) SOD. Values are means ± SE (*n* = 6). Different lowercase letters indicate significant differences between treatments (*p* < 0.05).

Antioxidant enzyme activities including APX, CAT, GPX, and SOD were investigated, and they showed contrapuntal responses to salinity stress. APX and GPX activity increased, but only at the highest salt concentration of 150 mM—increasing three- and four-fold, respectively (Figure 2A,B). CAT activity in contrast decreased under all saline treatment conditions (Figure 2C). SOD activity was higher at 25 and 50 mM NaCl compared to 100 mM of NaCl, while at 150 mM, enzyme activity was the same as the control treatment (Figure 2D). 

### 3.5. Relative Levels of Metabolites in Gac Seedling Leaf Concerning NaCl Concentration

**Figure 3:**
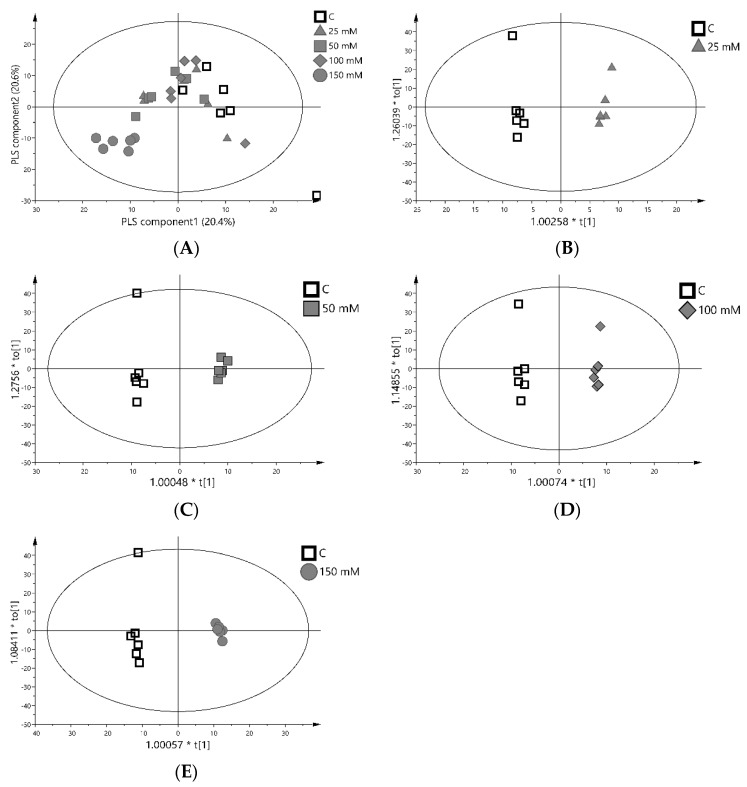
Multivariate analysis of metabolites assayed by GC−MS in gac leaf grown under salinity. (**A**) PLS−DA, (**B**) OPLS−DA of control and 25 mM groups, (**C**) OPLS−DA of control and 50 mM groups, (**D**) OPLS-DA of control and 100 mM groups and (**E**) OPLS−DA of control and 150 mM groups. Each of six biological replicates was individually plotted and the samples were projected onto a bi-plot showing the first two PCs. Each symbol on the plot represents data from 637 metabolites reduced to a single data point defined by the first (PLSC_1_) and second (PLSC_2_) PLSC. Samples that have similar metabolite compositions will cluster together while samples that are different will be further apart. The percentage variation explained by each PC is shown in parentheses on each axis. Please note the x-axes were automatically drawn to differing scales and do not allow for a true comparison of the distinction between control and salt treatment across experiments.

We determined the extent to which salt concentrations affected gac leaf polar GC-MS-TOF-generated metabolic profiles using multivariate analyses. On these plots, samples with similar metabolic profiles will group together, while those with distinct compositions will be more distant. There was no differentiation of samples by PCA analysis suggesting that the metabolite composition of the control and the salt-treated samples were similar (Figure S4). We, therefore, used the partial least squares discriminant analysis (PLS−DA), a supervised method, in an attempt to achieve better separation of the treatments [67,68]. There was no distinction between the control, 25 mM, 50 mM, and 100 mM samples, whereas the 150 mM group was significantly split from the others as shown in Figure 3A. OPLS-DA was used to find the difference between the two groups (control and each stress group) shown in Figure 3B–E. It shows a very clear separation between each paired sample; therefore, only the control and the 150 mM OPLS−DA model were valid [69].

**Figure 4:**
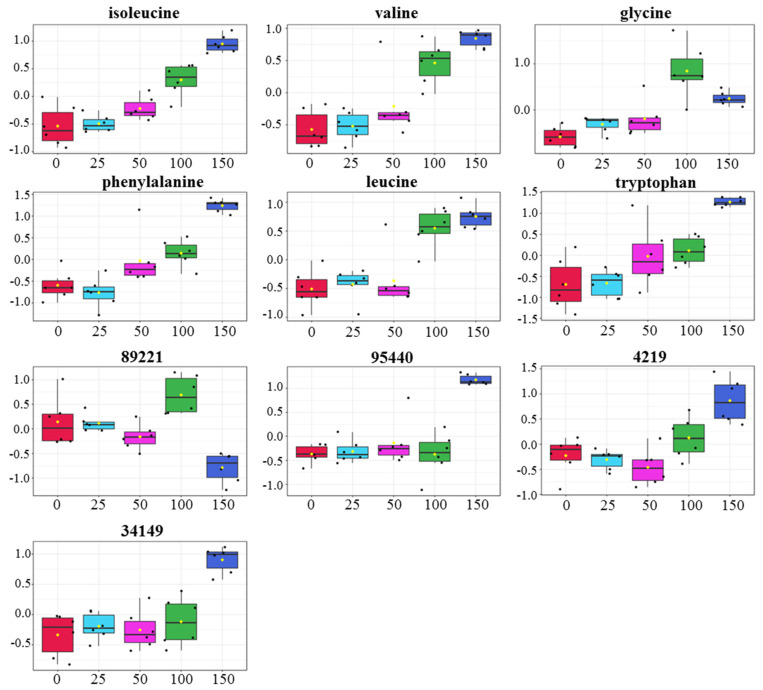
Metabolites that differed in gac samples due to salinity. One−way ANOVA analysis of significant metabolites measured by GC−MS−TOF in gac leaf grown under 0, 25, 50, 100, and 150 mM NaCl. Each metabolite (represented by a black dot) was individually plotted at the adjusted *p*−value = 0.05, post hoc analysis: Fisher’s LSD.

**Figure 5:**
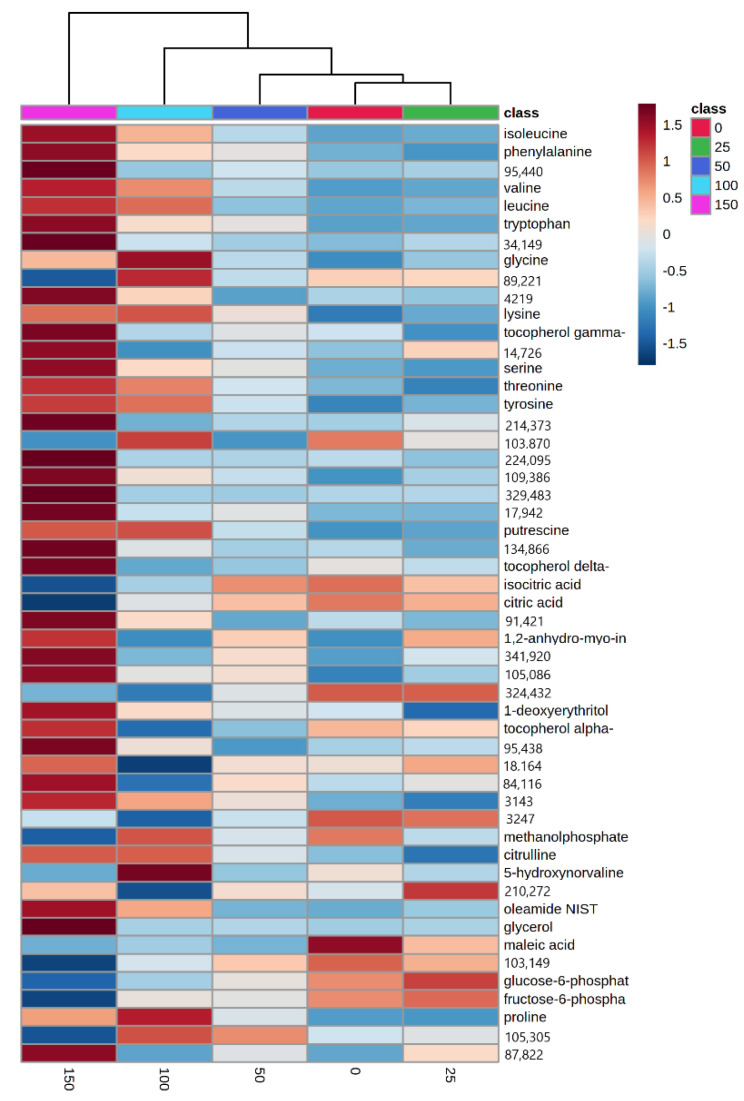
A heat map provides an intuitive visualization of metabolites found in this study. Each colored cell on the map corresponds to a concentration value in the data table, with samples in rows and metabolites in columns. Displayed are the top 52 metabolites ranked by *t*−tests with *p*−values by ANOVA of < 0.05 and FDR < 0.05.

To identify the metabolites in gac leaf that differed due to NaCl treatments, the mean of each metabolite was compared using one-way ANOVA analysis. Approximately 8% of the metabolites, i.e., 52 of the 637 metabolites differed (*p* value < 0.05 and FDR < 0.05; Figure S9). Using a more stringent *p* value, i.e., *p* < 10^−5^ as a cut-off, we found ten metabolites that differed, four of which were unknowns (‘95440’, ‘34149’, ‘89221’, and ‘4219’), while the others were all amino acids, i.e., valine, isoleucine, phenylalanine, leucine, tryptophan, and glycine (Figure 4). Interestingly, all amino acids except glycine increased due to the 150 mM salt treatment. A heat map depicting the 52 metabolites clearly showed that the 150 mM treatment was distinct from the others because of the drastic change in metabolite level. The heat map also showed that the ten highly differentiated metabolites by ANOVA also showed a similar pattern of change in response to salinity (Figure 5). 

**Figure 6:**
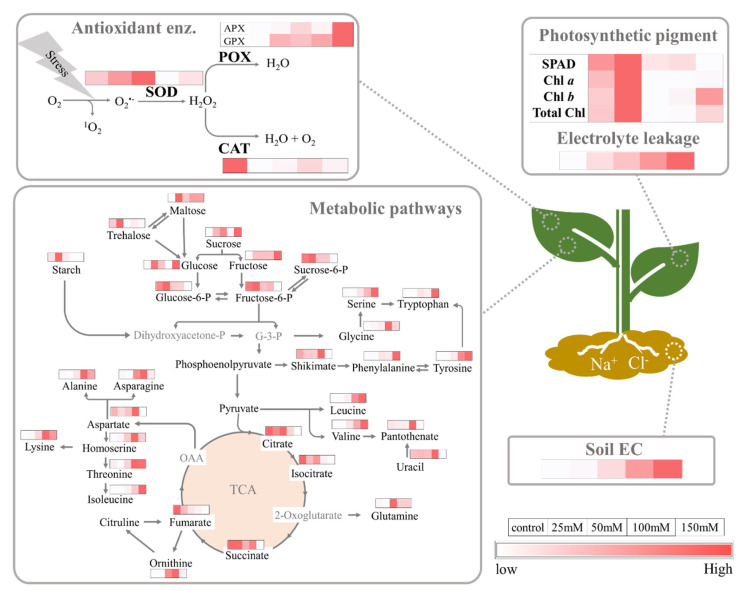
A Schematic showing the coordinate biochemical and physiological changes in gac seedling leaves in response to salinity. Shown are the relative metabolite levels, antioxidant enzyme activities, chlorophyll content and electrolyte leakage in response to increasing soil electrical conductivity. Arrows indicate the direction of the reaction. Heat maps showing the difference in each parameter under 0, 25, 50, 100, and 150 mM. Parameters in white boxes are present at relative low levels, while those in darker red boxes are higher.

We next sought to determine the specific metabolites that differed from the control in the plants grown under each saline condition. The fold−change analysis revealed that 27, 43, 47, and 205 metabolites differed significantly at 25, 50, 100, and 150 mM NaCl, (*p* < 0.05), respectively, when compared to the control (Figure S8 and Table S2). To obtain an overview of the similarities and/or differences of the identified metabolites, the behavior of the identified metabolites was analyzed using pathway impact. Pathway impact scores provide a measure of the importance of a given pathway relative to others assessed (See Liu et al., 2019 for additional details). Pathway analyses were conducted using the MetaboAnalyst 5.0 computational platform to better understand the impact of salinity levels on gac metabolic profiles [65,66,67]. A single *p*-value for each metabolic pathway was computed by pathway enrichment analysis, as opposed to a *t*-test [70]. The results showed that there was no pathway impact in the 50 mM treatment, i.e., there were no metabolic pathways enriched or distinguishable from others due to this treatment. In the 25 mM−treated plants, two pathways (*p*-values < 0.05) were enriched, however, the FDR values were too high (0.66353), so they were not considered (Table S3). There were nine pathways of identified metabolites with higher impact (*p* < 0.05) when the 100 mM and 150 mM treatments were examined (Figures S6 and S7). The metabolites were shown in the schematic metabolic pathway (Figure 6). At mild stress, many carbohydrates including starch, trehalose, maltose, sucrose-6-P, glucose-6-P, fructose-6-P, and TCA substances increased or remained the same as the control, then decreased in higher NaCl concentration, while for amino acids, the opposite trend was found.

## 4. Discussion

The aim of this study was to investigate key physiological and metabolic responses of gac to salinity, including potential acclimation, resistance, and exhaustion responses. Understanding the dynamics of plant response to salinity would help to develop solutions for more resilient plants, either through cultural practices or plant breeding. Saline soils are classified as those having an EC value of 4000 µScm^−1^ or more [71,72,73], but many crops are sensitive to EC values lower than 4000 µScm^−1^ [74]. In this study, the NaCl corresponded to 800 to 5000 µScm^−1^ which spans low (25–50 mM) to moderate (100–150 mM) salinity stress levels [75,76] as shown in Figure S1. Electrolyte leakage is used as a proxy for stress-induced injury and tolerance, as it often co-occurs with other stress physiological traits [62,77,78,79]. Electrolyte leakage was only impaired at higher NaCl levels, i.e., at 100 and 150 mM (Figure 1A), indicating that there were adaptative mechanisms at 25 and 50 mM. It was therefore of interest to identify biochemical changes that differed at these time points. At low salinity stress, we found that there was carbohydrate accumulation that may function in salt tolerance by playing an osmoprotective, and ROS scavenging role [80]. At a moderate level, many amino acids were increased (Figure 6).

*Antioxidant activity.* Salinity stress induces electrolyte leakage which in turn, is associated with ROS accumulation [81,82], and cellular oxidative damage [83,84]. APX, CAT, GPX, and SOD, are often deployed to detoxify ROS and engender stress tolerance [33,36,85,86,87,88]. The relative proportion of these enzymes determines the intracellular levels of ROS, and changes in their relative balance may induce stress tolerance mechanisms [89]. SOD is the first line of defense against ROS by catalyzing the dismutation of O_2_ to H_2_O_2_ [90,91]. SOD activity increased when the seedlings were exposed to moderate salinity (Figure 2D), but decreased at high salinity. Similar results were seen in *Brassica napus* L., where 100 to 150 mM NaCl decreased SOD activity [92]. ROS can be generated by CAT, which is responsible for the removal of H_2_O_2_ by reducing H_2_O_2_ to 2H_2_O. Our observations also strongly imply, that SOD, APX, and GPX enzymes are the salinity-induced ROS scavenging mechanisms in gac seedling leaves. Moreover, increased plant antioxidants is positively associated with decreased oxidative damage and improved salinity tolerance [93,94,95,96]. From the heat map (Figure 5), putrescine increased at 100 and 150 mM NaCl. Zhang et al. [97] reported that putrescine plays a positive role in salt-tolerance mechanisms by reducing oxidative damage in soybean. Jiménez-Bremont et al. also reported that as free putrescine increased in maize leaves oxidative damage during NaCl stress decreased [98]. Tang and Newton [99] proposed that exogenous putrescine could improve plant growth under NaCl stress, by inhibiting Na^+^ and Cl^−^ uptake, and accelerating the accumulation of K^+^, Ca^2+^, and Mg^2+^ in the leaves, thereby protecting plants from salt stress damage.

Leaf chlorophyll content. High salt interferes with leaf photosynthetic machinery which can be manifested as reduced chlorophyll. Our results showed a small reduction in chlorophyll a, and SPAD values when gac seedlings were subjected to 50, 100, and 150 mM NaCl, which agrees with previous reports [100,101]. Salinity−induced decreases in chlorophyll levels is associated with thylakoid membrane degradation, increased chlorophyllase activity, and inhibition of magnesium-protoporphyrin IX monomethyl ester (oxidative) cyclase due to oxidative stress [102,103,104,105,106]. In our study, salinity affected chlorophyll a but not chlorophyll b content, an observation made in other studies [107,108]. Chlorophyll *b* can be synthesized from chlorophyll a [109]. In *Cymbopogon nardus*, chlorophyll *a* appears to be more sensitive than chlorophyll b to salinity [110] and salinity could trigger chlorophyll a conversion to chlorophyll b. Interestingly, chlorophyll a, total chlorophyll, and SPAD value of gac seedling leaf exposed to 25 mM NaCl were increased which were supported by Elhaak et al. [111], Gomes et al. [112], and Gong et al. [108]. 

Although photosynthetic pigments decreased when gac seedlings were exposed to 150 mM NaCl, the heat map results (Figure 5) showed an increase in 1-deoxyerythriol which is involved in the biosynthesis of chlorophyll side chains [113]. Gamma, delta, and alpha-tocopherol, which are in chloroplasts and which act as antioxidants, also increased [114]. Salt stress also increases plants’ respiration rate due to a need for higher energy for adaptive mechanisms such as ion exclusion, compatible solute synthesis, and ROS detoxification [115]. In wheat, higher NaCl concentration increased the respiration rate [116], moreover, more and ATP was higher in a salt-tolerant wheat cultivar compared to a salt-sensitive one [117,118]. Therefore, plants may show tolerance to salinity by increasing photosynthetic pigments for higher photosynthetic capacity and ATP production. The resistance of photosynthetic systems to salinity is associated with the ability of the plant to effectively sequester the ions in the chloroplast and the other organelles [119]. 

Carbohydrate metabolism plays an important role as an adaptive response to salinity [120,121,122,123,124] and it also has been associated with salinity-tolerant mechanisms in many plants, via osmotic adjustments [125]. Accumulation of sugars as compatible solutes is accompanied by the influx of water into a plant cell [126]. Exposure to 25 mM NaCl triggered higher starch content and was also associated with the accumulation of some photosynthetic pigments (Figure 1D and Figure S3). It is possible to speculate that higher photosynthetic activity was linked to an increase in starch content at low salinity. Moreover, starch increased at 25 mM and total soluble sugar content at 50 mM NaCl compared to the control and the other groups. Pattanagul and Thitisaksakul [46] also reported that sugar content was induced by salinity stress in salt-sensitive rice as an osmotic adjustment. Therefore, these results agree with the previous studies on the responses of plants under salinity stress [127,128]. Leaf starch may hyperaccumulate to prevent feedback inhibition of photosynthesis and then under prolonged stress act as a store of sugars for osmoprotection [127]. In addition to carbohydrate accumulation, there were increases in other compounds that serve as osmoprotectants: glycine, 5-hydroxynorvaline, glucose-6-phosphate, fructose-6-phosphate, and proline [129,130,131,132,133]. 

Our study found that six metabolites were significantly decreased when gac seedlings were grown under salinity conditions. These metabolites included isocitric acid, citric acid [134], and maleic acid [135], which are found in the TCA cycle (Figure 6). In a study of salt-treated wheat [116], higher respiration rates and extensive metabolite changes were shown. The activity of key enzymes of the TCA cycle was shown to be directly salt-sensitive by increased GABA shunt activity, which provides an alternative carbon source for mitochondria that bypasses salt-sensitive enzymes, to facilitate increased respiration [116]. In accordance with our carbohydrate metabolism results, high salinity increased starch degradation and sucrose and glucose, and fructose content, which could enhance flux through the respiratory pathways and increase ATP. However, if key enzymes of the TCA cycle are inhibited, then it is unlikely that ATP would be generated. 

Lysine and citrulline are important indicators of salt tolerance, and both increased in gac seedling leaves, [135,136]. Under high salinity (150 mM), there are 33 metabolic changes, which could be a response to the cellular damage induced by higher NaCl concentrations [137,138], precursors of IAA [139,140], compatible solutes protecting cells from osmotic stress [141,142,143,144], precursors of biosynthesis of photosynthetic pigments [114], antioxidant molecules in chloroplasts [114].

Pathway impact was used to identify metabolic pathway associated with changes in salinity. Many metabolic pathways were altered, especially amino acid metabolism pathways, which have roles in stress response [141,145,146,147,148,149,150,151,152,153,154]. Our study found that many organic acids including TCA cycle intermediates such as pyruvic acid, oxalic acid, malic acid, citric acid, and succinic acid declined under salinity. The reduction in organic acids, and TCA cycle intermediates may be due to the diversion of carbon flux towards the synthesis of osmoprotectants to cope with salt stress [155,156]. Salinity-induced reductions in organic acids are a metabolic phenotype reported in halophytes such as *Thellungiella halophila, Suaeda salsa*, *Salvadora persica,* and *Chenopodium quinoa* [155,156,157]. Under salt stress, the regulation of organic acid metabolism is intimately related to growth reduction, and it might be an energy-saving strategy by inducing changes in starch metabolism and sugar export in the source [158]. Salinity stress at 100 mM affected glucosinolate, lysine biosynthesis [159], cyanoamino acid metabolism [160], and valine, leucine, and isoleucine degradation (Figure S6). However, higher salinity stress had no impact on these metabolic pathways. Our study also found that starch and sucrose, galactose, and *β*-alanine metabolism were changed when gac seedlings were grown under 150 mM NaCl (Figure S7). Sugar and sugar derivatives found in these pathways are likely an adaptive response that helps plants cope with water stress via an osmotic mechanism, energy metabolism, and ROS scavenging. 

## 5. Conclusions

**Figure 7:**
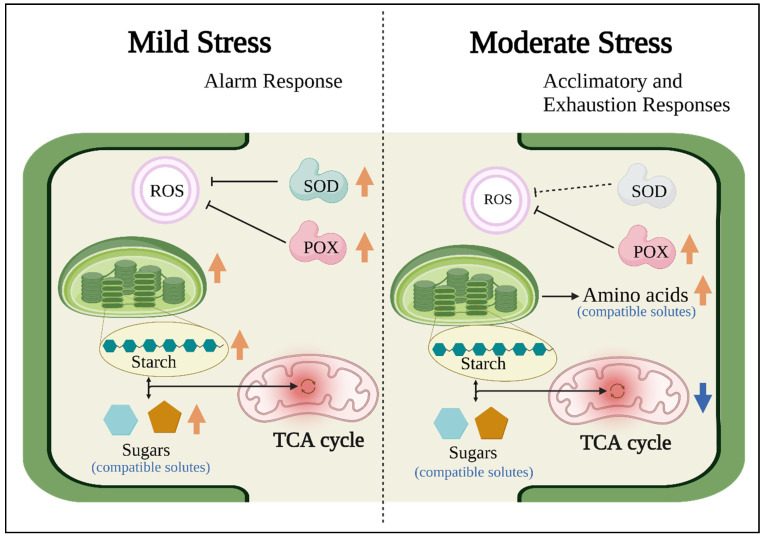
A Schematic comparing mild salinity stress and moderate salinity stress responses of gac plant. Figure is based on data generated in this study.

This study observed changes in physiological characteristics and metabolic profiles in response to mild and moderate salinity stress in gac leaf. At mild stress, which may be the alarm response, SOD and POX enzyme activity increased and were probably associated with ROS scavenging in stress tolerance (Figure 7). Photosynthetic pigments slightly increased presumably for maintaining energy stores (Figure 7). Increases in starch may prevent feedback inhibition of photosynthesis and then, serve as a store of sugars for osmoprotection (Figure 7). Higher salt concentrations revealed the acclimatory and exhaustion responses, POX enzymes were still active while SOD enzymes decreased. Moreover, metabolites in the TCA cycle decreased, presumably because of a greater flux of carbon towards amino acids production, to act as compatible solutes. Further research should focus on the mechanisms regulating the level of metabolites involved in salinity stress tolerance, especially amino acids for the determination of key pathways controlling high salinity tolerance in plants.

## Data Availability

Data made available on request.

## Supplementary Materials

The following supporting information can be downloaded at: https://www.mdpi.com/article/10.3390/plants11192447/s1 Figure S1: Soil electrical conductivity; Figure S2: Scatter plot of electrolyte leakage vs. soil EC; Figure S3: Assessment of chlorophyll by SPAD in leaves of salinity-treated gac seedlings; Figure S4: PCA score plots Figure S5: PCA scree plots; Figure S6: Pathway impact metabolic changes compared to control found in 100 mM; Figure S7: Pathway impact metabolic changes compared to control found in 150 mM; Figure S8: Volcano plots of significant metabolites in each treatment compared to control; Figure S9: One-way ANOVA analysis; Table S1: List of 52 significantly variable metabolites; Table S2: List of 205 metabolites; Table S3: Pathway impact list.

## References

[1] Ishida B.K., Turner C., Chapman M.H., McKeon T.A. (2004). Fatty Acid and Carotenoid Composition of Gac (*Momordica Cochinchinensis* Spreng) Fruit. J. Agric. Food Chem..

[2] Aoki H., Kieu N.T.M., Kuze N., Tomizaka K., Chuyen N.V. (2002). Carotenoid Pigments in GAC Fruit ( *Momordica Cochinchinensis* SPRENG). Biosci. Biotechnol. Biochem..

[3] Yu J.S., Roh H.-S., Lee S., Jung K., Baek K.-H., Kim K.H. (2017). Antiproliferative Effect of *Momordica Cochinchinensis* Seeds on Human Lung Cancer Cells and Isolation of the Major Constituents. Rev. Bras. Farmacogn..

[4] Vuong L.T., Dueker S.R., Murphy S.P. (2002). Plasma Beta-Carotene and Retinol Concentrations of Children Increase after a 30-d Supplementation with the Fruit *Momordica Cochinchinensis* (Gac). Am. J. Clin. Nutr..

[5] Osganian S.K., Stampfer M.J., Rimm E., Spiegelman D., Manson J.E., Willett W.C. (2003). Dietary Carotenoids and Risk of Coronary Artery Disease in Women. Am. J. Clin. Nutr..

[6] Nagarani G., Abirami A., Siddhuraju P. (2014). Food Prospects and Nutraceutical Attributes of *Momordica* Species: A Potential Tropical Bioresources—A Review. Food Sci. Hum. Wellness.

[7] Mai C.H., Truong V., Frederic D. (2013). Optimisation of Enzyme-Assisted Extraction of Oil Rich in Carotenoids from Gac Fruit (*Momordica Cochinchinensis* Spreng.). Food Technol. Biotechnol..

[8] Kubola J., Siriamornpun S. (2011). Phytochemicals and Antioxidant Activity of Different Fruit Fractions (Peel, Pulp, Aril and Seed) of Thai Gac (*Momordica Cochinchinensis* Spreng). Food Chem..

[9] Zhao L.M., Han L.N., Ren F.Z., Chen S.H., Liu L.H., Wang M.X., Sang M.X., Shan B.E. (2012). An Ester Extract of Cochinchina Momordica Seeds Induces Differentiation of Melanoma B16 F1 Cells via MAPKs Signaling. Asian Pacific J. Cancer Prev..

[10] Meng L.-Y., Liu H.-R., Shen Y., Yu Y.-Q., Tao X. (2011). Cochinchina Momordica Seed Extract Induces G2/M Arrest and Apoptosis in Human Breast Cancer MDA-MB-231 Cells by Modulating the PI3K/Akt Pathway. Asian Pac. J. Cancer Prev..

[11] Liu D., Ford K.L., Roessner U., Natera S., Cassin A.M., Patterson J.H., Bacic A. (2013). Rice Suspension Cultured Cells Are Evaluated as a Model System to Study Salt Responsive Networks in Plants Using a Combined Proteomic and Metabolomic Profiling Approach. Proteomics.

[12] Shannon M.C., Grieve C.M. (1998). Tolerance of Vegetable Crops to Salinity. Sci. Hortic..

[13] Bacilio M., Rodriguez H., Moreno M., Hernandez J.-P., Bashan Y. (2004). Mitigation of Salt Stress in Wheat Seedlings by a Gfp-Tagged *Azospirillum lipoferum*. Biol. Fertil. Soils.

[14] Ashraf M., Foolad M.R. (2007). Roles of Glycine Betaine and Proline in Improving Plant Abiotic Stress Resistance. Environ. Exp. Bot..

[15] Yan N., Marschner P., Cao W., Zuo C., Qin W. (2015). Influence of Salinity and Water Content on Soil Microorganisms. Int. Soil Water Conserv. Res..

[16] George R., McFarlane D., Nulsen B. (1997). Salinity Threatens the Viability of Agriculture and Ecosystems in Western Australia. Hydrogeol. J..

[17] Akbarimoghaddam H., Galavi M., Ghanbari A., Panjehkeh N. (2011). Salinity Effects on Seed Germination and Seedling Growth of Bread Wheat Cultivars. Trakia J. Sci..

[18] Asada K. (2006). Production and Scavenging of Reactive Oxygen Species in Chloroplasts and Their Functions. Plant Physiol..

[19] Benzarti M., Rejeb K.B., Messedi D., Mna A.B., Hessini K., Ksontini M., Abdelly C., Debez A. (2014). Effect of High Salinity on *Atriplex portulacoides*: Growth, Leaf Water Relations and Solute Accumulation in Relation with Osmotic Adjustment. South Afr. J. Bot..

[20] James R.A., Blake C., Byrt C.S., Munns R. (2011). Major Genes for Na^+^ Exclusion, Nax1 and Nax2 (Wheat HKT1;4 and HKT1;5), Decrease Na^+^ Accumulation in Bread Wheat Leaves under Saline and Waterlogged Conditions. J. Exp. Bot..

[21] Munns R., James R.A., Läuchli A. (2006). Approaches to Increasing the Salt Tolerance of Wheat and Other Cereals. J. Exp. Bot..

[22] Yasar F., Kusvuran S., Ellialtioglu S. (2006). Determination of Anti-Oxidant Activities in Some Melon (*Cucumis melo* L.) Varieties and Cultivars under Salt Stress. J. Hortic. Sci. Biotechnol..

[23] Rady M.M., Taha R.S., Mahdi A.H.A. (2016). Proline Enhances Growth, Productivity and Anatomy of Two Varieties of *Lupinus termis* L. Grown under Salt Stress. South Afr. J. Bot..

[24] Hadjigogos K. (2003). The Role of Free Radicals in the Pathogenesis of Rheumatoid Arthritis. Panminerva Med..

[25] Yen W.-J., Chyau C.-C., Lee C.-P., Chu H.-L., Chang L.-W., Duh P.-D. (2013). Cytoprotective Effect of White Tea against H2O2-Induced Oxidative Stress in Vitro. Food Chem..

[26] Hussain T.M., Chandrasekhar T., Hazara M., Sultan Z., Saleh B.K., Gopal G.R. (2008). Recent Advances in Salt Stress Biology—A Review. Biotechnol. Mol. Biol. Rev..

[27] Gill S.S., Tuteja N. (2010). Reactive Oxygen Species and Antioxidant Machinery in Abiotic Stress Tolerance in Crop Plants. Plant Physiol. Biochem..

[28] Karuppanapandian T., Moon J.C., Kim C., Manoharan K., Kim W. (2011). Reactive Oxygen Species in Plants: Their Generation, Signal Transduction, and Scavenging Mechanisms. Aust. J. Crop Sci..

[29] Erofeeva E.A. (2015). Dependence of Guaiacol Peroxidase Activity and Lipid Peroxidation Rate in Drooping Birch (*Betula Pendula* Roth) and Tillet (*Tilia Cordata* Mill) Leaf on Motor Traffic Pollution Intensity. Dose-Response.

[30] Noctor G., Foyer C.H. (1998). Ascorbate and Glutathione: Keeping Active Oxygen Under Control. Annu. Rev. Plant Physiol. Plant Mol. Biol..

[31] Asada K. (1999). The Water-Water Cycle in Chloroplasts: Scavenging of Active Oxygens and Dissipation of Excess Photons. Annu. Rev. Plant Biol..

[32] Niyogi K.K. (1999). Photoprotection Revisited: Genetic and Molecular Approaches. Annu. Rev. Plant Physiol. Plant Mol. Biol..

[33] You J., Chan Z. (2015). ROS Regulation During Abiotic Stress Responses in Crop Plants. Front. Plant Sci..

[34] Shulaev V., Cortes D., Miller G., Mittler R. (2008). Metabolomics for Plant Stress Response. Physiol. Plant..

[35] Obata T., Fernie A.R. (2012). The Use of Metabolomics to Dissect Plant Responses to Abiotic Stresses. Cell. Mol. Life Sci..

[36] Arbona V., Manzi M., de Ollas C., Gómez-Cadenas A. (2013). Metabolomics as a Tool to Investigate Abiotic Stress Tolerance in Plants. Int. J. Mol. Sci..

[37] Munns R., Termaat A. (1986). Whole-Plant Responses to Salinity. Aust. J. Plant Physiol..

[38] Delauney A.J., Verma D.P.S. (1993). Proline Biosynthesis and Osmoregulation in Plants. Plant J..

[39] Flowers T.J., Flowers S.A. (2005). Why Does Salinity Pose Such a Difficult Problem for Plant Breeders?. Agric. Water Manag..

[40] Paul D. (2013). Osmotic Stress Adaptations in Rhizobacteria. J. Basic Microbiol..

[41] Dasgan H.Y., Balacheva E., Yetişir H., Yarsi G., Altuntas O., Akhoundnejad Y., Coban A. (2015). The Effectiveness of Grafting to Improve Salt Tolerance of Sensitive Melon When the Tolerant Melon Is Use as Rootstock. Procedia Environ. Sci..

[42] Ford C.W. (1984). Accumulation of Low Molecular Weight Solutes in Water-Stressed Tropical Legumes. Phytochemistry.

[43] Bressan R.A., Hasegawa P.M., Pardo J.M. (1998). Plants Use Calcium to Resolve Salt Stress. Trends Plant Sci..

[44] Dubey R.S., Singh A.K. (1999). Salinity Induces Accumulation of Soluble Sugars and Alters the Activity of Sugar Metabolising Enzymes in Rice Plants. Biologia Plant..

[45] Flowers T.J. (2004). Improving Crop Salt Tolerance. J. Exp. Bot..

[46] Pattanagul W., Thitisaksakul M. (2008). Effect of Salinity Stress on Growth and Carbohydrate Metabolism in Three Rice *(Oryza Sativa* L.) Cultivars Differing in Salinity Tolerance. Ind. J. Exp. Bio..

[47] Amirjani M.R. (2011). Effect of Salinity Stress on Growth, Sugar Content, Pigments and Enzyme Activity of Rice. Int. J. Bot..

[48] Yin Y.G., Kobayashi Y., Sanuki A., Kondo S., Fukuda N., Ezura H., Sugaya S., Matsukura C. (2010). Salinity Induces Carbohydrate Accumulation and Sugar-Regulated Starch Biosynthetic Genes in Tomato (*Solanum lycopersicum* L. Cv. ‘Micro-Tom’) Fruits in an ABA- and Osmotic Stress-Independent Manner. J. Exp. Bot..

[49] Nemati I., Moradi F., Gholizadeh S., Esmaeili M.A., Bihamta M.R. (2011). The Effect of Salinity Stress on Ions and Soluble Sugars Distribution in Leaves, Leaf Sheaths and Roots of Rice (*Oryza Sativa* L.) Seedlings. Plant Soil Environ..

[50] Roberts M.F. (2005). Organic Compatible Solutes of Halotolerant and Halophilic Microorganisms. Saline Syst..

[51] Jumpa T., Pattanagul W., Songsri P. (2017). Effects of Salinity Stress on Some Physiological Traits in Gac (*Momordica Cochinchinensis* (Lour.) Spreng.). Khon Kaen Agric. J..

[52] Robbins N.S., Pharr D.M. (1987). Regulation of Photosynthetic Carbon Metabolism in Cucumber by Light Intencity and Photosynthetic Period. Plant Physiol..

[53] Madore M.A. (1990). Carbohydrate Metabolism in Photosynthetic and Nonphotosynthetic Tissues of Variegated Leaves of *Coleus blumei* Benth. Plant Physiol..

[54] Lu S., Su W., Li H., Guo Z. (2009). Abscisic Acid Improves Drought Tolerance of Triploid ermudagrass and Involves H_2_O_2_- and NO-Induced Antioxidant Enzyme Activities. Plant Physiol. Biochem..

[55] Bradford M.M. (1976). A Rapid and Sensitive Method for the Quantitation of Microgram Quantities of Protein Utilizing the Principle of Protein-Dye Binding. Anal. Biochem..

[56] Sun Z., Henson C.A. (1991). A Quantitative Assessment of the Importance of Barley Seed α-Amylase, β-Amylase, Debranching Enzyme, and α-Glucosidase in Starch Degradation. Arch. Biochem. Biophys..

[57] Chandlee J.M., Scandalios J.G. (1984). Analysis of Variants Affecting the Catalase Developmental Program in Maize Scutellum. Theor. Appl. Genet..

[58] Nakano Y., Asada K. (1981). Hydrogen Peroxide Is Scavenged by Ascorbate-Specific Peroxidase in Spinach Chloroplasts. Plant Cell Physiol..

[59] Arnon D.I. (1949). Copper Enzymes in Isolated Chloroplasts Polyphenoloxidase in *Beta vulgaris*. Plant. Physiol..

[60] Ling Q., Huang W., Jarvis P. (2011). Use of a SPAD-502 Meter to Measure Leaf Chlorophyll Concentration in *Arabidopsis thaliana*. Photosynth. Res..

[61] Yuan Z., Cao Q., Zhang K., Ata-Ul-Karim S.T., Tian Y., Zhu Y., Cao W., Liu X. (2016). Optimal Leaf Positions for SPAD Meter Measurement in Rice. Front. Plant Sci..

[62] Baninasab B., Ghobadi C. (2011). Influence of Paclobutrazol and Application Methods on High-Temperature Stress Injury in Cucumber Seedlings. J. Plant Growth Regul..

[63] Fiehn O., Wohlgemuth G., Scholz M., Kind T., Lee D.Y., Lu Y., Moon S., Nikolau B. (2008). Quality Control for Plant Metabolomics: Reporting MSI-Compliant Studies. Plant J..

[64] Xia J., Sinelnikov I.V., Han B., Wishart D.S. (2015). MetaboAnalyst 3.0—Making Metabolomics More Meaningful. Nucleic Acids Res..

[65] Xia J., Wishart D.S. (2016). Using MetaboAnalyst 3.0 for Comprehensive Metabolomics Data Analysis. Curr. Protoc. Bioinform..

[66] Chong J., Soufan O., Li C., Caraus I., Li S., Bourque G., Wishart D.S., Xia J. (2018). MetaboAnalyst 4.0: Towards More Transparent and Integrative Metabolomics Analysis. Nucleic Acids Res..

[67] Kermit M., Tomic O. (2003). Independent Component Analysis Applied on Gas Sensor Array Measurement Data. IEEE Sens. J..

[68] Scholz M., Gatzek S., Sterling A., Fiehn O., Selbig J. (2004). Metabolite Fingerprinting: Detecting Biological Features by Independent Component Analysis. Bioinformatics.

[69] Worley B., Powers R. (2016). PCA as a Practical Indicator of OPLS-DA Model Reliability. Curr. Metab..

[70] Liu G., Lee D.P., Schmidt E., Prasad G.L. (2019). Pathway Analysis of Global Metabolomic Profiles Identified Enrichment of Caffeine, Energy, and Arginine Metabolism in Smokers but Not Moist Snuff. Bioinform. Biol. Insights.

[71] Richards L.A. (1954). Diagnosis and Improvement of Saline and Alkaline Soils. Soil Sci. Soc. Am. J..

[72] Qadir M., Schubert S., Steffens D. (2005). Phytotoxic Substances in Soils. Encyclopedia of Soils in the Environment.

[73] Paul B.K., Rashid H., Paul B.K., Rashid H. (2017). Nonstructural Adaptation. Climimatic Hazards in Coastal Bangladesh.

[74] Gupta B., Huang B. (2014). Mechanism of Salinity Tolerance in Plants: Physiological, Biochemical, and Molecular Characterization. Int. J. Genom..

[75] Im-Erb R., Neawsuparb K., Sombatpanit S. (2013). Soil Salinization Assessment and Monitoring at Boe Klue District, Nan Province, Northern Thailand. Developments in Soil Salinity Assessment and Reclamation: Innovative Thinking and Use of Marginal Soil and Water Resources in Irrigated Agriculture.

[76] Arunin S., Pongwichian P. (2015). Salt-Affected Soils and Management in Thailand. Bull. Soc. Sea Water Sci. Japan.

[77] Ambede J.G., Netondo G.W., Mwai G.N., Musyimi D.M. (2012). NaCl Salinity Affects Germination, Growth, Physiology, and Biochemistry of Bambara Groundnut. Brazilian J. Plant Physiol..

[78] Abreu I.A., Farinha A.P., Negrão S., Gonçalves N., Fonseca C., Rodrigues M., Batista R., Saibo N.J.M., Oliveira M.M. (2013). Coping with Abiotic Stress: Proteome Changes for Crop Improvement. J. Proteom..

[79] Sohrabi S., Ebadi A., Jalali S., Salami S.A. (2017). Enhanced Values of Various Physiological Traits and VvNAC1 Gene Expression Showing Better Salinity Stress Tolerance in Some Grapevine Cultivars as Well as Rootstocks. Sci. Hortic..

[80] Gil R., Boscaiu M., Lull C., Bautista I., Lidón A., Vicente O. (2013). Are Soluble Carbohydrates Ecologically Relevant for Salt Tolerance in Halophytes?. Funct. Plant Biol..

[81] Apel K., Hirt H. (2004). Reactive Oxigen Species: Metabolism, Oxidative Stress, and Signal Transduction. Annu. Rev. Plant Biol..

[82] Demidchik V., Straltsova D., Medvedev S.S., Pozhvanov G.A., Sokolik A., Yurin V. (2014). Stress-Induced Electrolyte Leakage: The Role of K^+^-Permeable Channels and Involvement in Programmed Cell Death and Metabolic Adjustment. J. Exp. Bot..

[83] Bailey-Serres J. (2006). The Roles of Reactive Oxygen Species in Plant Cells. Plant Physiol..

[84] Caverzan A., Casassola A., Patussi Brammer S. (2016). Reactive Oxygen Species and Antioxidant Enzymes Involved in Plant Tolerance to Stress. Abiotic and Biotic Stress in Plants—Recent Advances and Future Perspectives.

[85] Ahmad P., Ashraf M., Hakeem K.R., Azooz M., Rasool S., Chandna R., Akram N.A. (2014). Potassium Starvation-Induced Oxidative Stress and Antioxidant Defense Responses in *Brassica Juncea*. J. Plant Interact..

[86] Mika A., Lüthje S. (2003). Properties of Guaiacol Peroxidase Activities Isolated from Corn Root Plasma Membranes. Plant Physiol..

[87] Passardi F., Longet D., Penel C., Dunand C. (2004). The Class III Peroxidase Multigenic Family in Rice and Its Evolution in Land. Plants Phytochemistry.

[88] Hanin M., Ebel C., Ngom M., Laplaze L., Masmoudi K. (2016). New Insights on Plant Salt Tolerance Mechanisms and Their Potential Use for Breeding. Front. Plant Sci..

[89] Mittler R. (2002). Oxidative Stress, Antioxidants and Stress Tolerance. Trends Plant Sci..

[90] Menezes-Benavente L., Teixeira F.K., Alvim Kamei C.L., Margis-Pinheiro M. (2004). Salt Stress Induces Altered Expression of Genes Encoding Antioxidant Enzymes in Seedlings of a Brazilian Indica Rice (*Oryza sativa* L.). Plant Sci..

[91] Scandalios J.G. (2005). Oxidative Stress: Molecular Perception and Transduction of Signals Triggering Antioxidant Gene Defenses. Brazilian J. Med. Biol. Res. = Rev. Bras. Pesqui. medicas e Biol..

[92] Zaefizadeh M., Mohammad S., Jalali-E-Emam S., Alizadeh B., Zakarya R.A., Khayatnezhad M. (2011). Superoxide Dismutase (SOD) Activity in Nacl Stress in Salt-Sensitive and Salt-Tolerance Genotypes of Colza (*Brassica napus* L.). Middle-East J. Sci. Res..

[93] Hasegawa P.M., Bressan R.A., Zhu J.-K., Bohnert H.J. (2000). Plant Cellular and Molecular and Molecular Responses to High Salinity. Annu. Rev. Plant Physiol. Plant Mol. Biol..

[94] Mittova V., Tal M., Volokita M., Guy M. (2003). Up-Regulation of the Leaf Mitochondrial and Peroxisomal Antioxidative Systems in Response to Salt-Induced Oxidative Stress in the Wild Salt-Tolerant Tomato Species *Lycopersicon pennellii*. Plant. Cell Environ..

[95] Demiral T., Turkan I. (2005). Comparative Lipid Peroxidation, Antioxidant Defense Systems and Proline Content in Roots of Two Rice Cultivars Differing in Salt Tolerance. Environ. Exp. Bot..

[96] El-Shabrawi H., Kumar B., Kaul T., Reddy M.K., Singla-Pareek S.L., Sopory S.K. (2010). Redox Homeostasis, Antioxidant Defense, and Methylglyoxal Detoxification as Markers for Salt Tolerance in Pokkali Rice. Protoplasma.

[97] Zhang G., Xu S., Hu Q., Mao W., Gong Y. (2014). Putrescine Plays a Positive Role in Salt-Tolerance Mechanisms by Reducing Oxidative Damage in Roots of Vegetable Soybean. J. Integr. Agric..

[98] Jiménez-Bremont J., Ruiz O., Rodríguez-Kessler M. (2007). Modulation of Spermidine and Spermine Levels in Maize Seedlings Subjected to Long-Term Salt Stress. Plant Physiol. Biochem. PPB.

[99] Tang W., Newton R.J. (2005). Polyamines Reduce Salt-Induced Oxidative Damage by Increasing the Activities of Antioxidant Enzymes and Decreasing Lipid Peroxidation in Virginia Pine. Plant Growth Regul..

[100] Turan M.A., Turkmen N., Nilgun T. (2007). Effect of NaCl on Stomatal Resistance and Proline, Chlorophyll, Na, Cl and K Concentrations of Lentil Plants. J. Agron..

[101] Taffouo V.D., Wamba O.F., Youmbi E., Nono G.V., Akoa A. (2009). Growth, Yield, Water Status and Ionic Distribution Response of Three Bambara Groundnut (*Vigna subterranea* (L.) Verdc.) Landraces Grown under Saline Conditions. Int. J. Bot..

[102] Aarti P.D., Tanaka R., Tanaka A. (2006). Effects of Oxidative Stress on Chlorophyll Biosynthesis in Cucumber (*Cucumis sativus*) Cotyledons. Physiol. Plant..

[103] Zhao G.Q., Ma B.L., Ren C.Z. (2007). Growth, Gas Exchange, Chlorophyll Fluorescence, and Ion Content of Naked Oat in Response to Salinity. Crop Sci..

[104] Ashraf M., Ali Q. (2008). Relative Membrane Permeability and Activities of Some Antioxidant Enzymes as the Key Determinants of Salt Tolerance in Canola (*Brassica napus* L.). Environ. Exp. Bot..

[105] Zhang H., Ye Y.K., Wang S.H., Luo J.P., Tang J., Ma D.F. (2009). Hydrogen Sulfide Counteracts Chlorophyll Loss in Sweetpotato Seedling Leaves and Alleviates Oxidative Damage against Osmotic Stress. Plant Growth Regul..

[106] Walid Z., Houneida A., Najoua M., Chayma O., Mokhtar L., Zeineb O. (2013). Photosynthetic Behaviour of *Arabidopsis thaliana* (Pa-1 Accession) under Salt Stress. African J. Biotechnol..

[107] Heidari M. (2012). Effects of Salinity Stress on Growth, Chlorophyll Content and Osmotic Components of Two Basil (*Ocimum basilicum* L.) Genotypes. African J. Biotechnol..

[108] Gong D.H., Wang G.Z., Si W.T., Zhou Y., Liu Z., Jia J. (2018). Effects of Salt Stress on Photosynthetic Pigments and Activity of Ribulose-1,5-Bisphosphate Carboxylase/Oxygenase in Kalidium Foliatum. Russ. J. Plant Physiol..

[109] Ito H., Ohtsuka T., Tanaka A. (1996). Conversion of Chlorophyll b to Chlorophyll a via 7-Hydroxymethyl Chlorophyll. J. Biol. Chem..

[110] Mane A.V., Karadge B.A., Samant J.S. (2010). Salinity Induced Changes in Photosynthetic Pigments and Polyphenols of *Cymbopogon nardus* (L.) Rendle. J. Chem. Pharm. Res..

[111] Elhaak M.A., Migahid M.M., Wegmann K. (2008). Response on Photosynthetic Pigments to Drought and Salt Stress in Some Desert Species. Feddes Repert..

[112] Gomes A.D.C., Pestana M., Abreu I., Claudete S.-C., Rachel Ann H.-D., Marina S. (2017). Salinity Effects on Photosynthetic Pigments, Proline, Biomass and Nitric Oxide in *Salvinia auriculata* Aubl. Orig. Artic. Acta Limnol. Bras..

[113] Wanke M., Skorupinska-Tudek K., Swiezewska E. (2001). Isoprenoid Biosynthesis via 1-Deoxy-D-Xylulose 5-Phosphate/2-C-Methyl-D-Erythritol 4-Phosphate (DOXP/MEP) Pathway. Acta Biochim. Pol..

[114] Munné-Bosch S. (2005). The Role of Alpha-Tocopherol in Plant Stress Tolerance. J. Plant Physiol..

[115] Che-Othman M., Jacoby R., Millar A., Taylor N. (2020). Wheat Mitochondrial Respiration Shifts from the Tricarboxylic Acid Cycle to the GABA Shunt under Salt Stress. New Phytol..

[116] Zidan M.A., Elewa M.A. (1995). Effect of Salinity on Germination, Seedling Growth and Some Metabolic Changes in Four Plant Species (*Umbelliferae*). Indian J. Plant Physiol..

[117] Ribas-Carbo M., Taylor N.L., Giles L., Busquets S., Finnegan P.M., Day D.A., Lambers H., Medrano H., Berry J.A., Flexas J. (2005). Effects of Water Stress on Respiration in Soybean Leaves. Plant Physiol..

[118] Shekoofa A., Bijanzadeh E., Emam Y., Pessarakli M. (2012). Effect of Salt Stress on Respipration of Various Wheat Lines/ Cultivars at Early Growth Stages. J. Plant Nutr..

[119] Wang H., Zhang M., Guo R., Shi D., Liu B., Lin X., Yang C. (2012). Effects of Salt Stress on Ion Balance and Nitrogen Metabolism of Old and Young Leaves in Rice (*Oryza Sativa* L.). BMC Plant Biol..

[120] Hoekstra F.A., Golovina E.A., Buitink J. (2001). Mechanisms of Plant Desiccation Tolerance. Trends Plant Sci..

[121] Seki M., Kamei A., Yamaguchi-Shinozaki K., Shinozaki K. (2003). Molecular Responses to Drought, Salinity and Frost: Common and Different Paths for Plant Protection. Curr. Opin. Biotechnol..

[122] Rolland F., Baena-Gonzalez E., Sheen J. (2006). Sugar Sensing and Signaling in Plants: Conserved and Novel Mechanisms. Annu. Rev. Plant Biol..

[123] Kempa S., Krasensky J., Dal Santo S., Kopka J., Jonak C. (2008). A Central Role of Abscisic Acid in Stress-Regulated Carbohydrate Metabolism. PLoS ONE.

[124] Farrant J.M., Moore J.P. (2011). Programming Desiccation-Tolerance: From Plants to Seeds to Resurrection Plants. Curr. Opin. Plant Biol..

[125] Gilbert G.A., Wilson C., Madore M.A. (1997). Root-Zone Salinity Alters Raffinose Oligosaccharide Metabolism and Transport in Coleus. Plant Physiol..

[126] Hare P.D., Cress W.A., Van Staden J. (1998). Dissecting the Roles of Osmolyte Accumulation during Stress. Plant Cell Environ..

[127] Dong S., Beckles D.M. (2019). Dynamic Changes in the Starch-Sugar Interconversion within Plant Source and Sink Tissues Promote a Better Abiotic Stress Response. J. Plant Physiol..

[128] Thalmann M., Santelia D. (2017). Starch as a Determinant of Plant Fitness under Abiotic Stress. New Phytol..

[129] Zhang H., Dong H., Li W., Sun Y., Chen S., Kong X. (2009). Increased Glycine Betaine Synthesis and Salinity Tolerance in AhCMO Transgenic Cotton Lines. Mol. Breed..

[130] Bagdi D.L., Shaw B.P. (2013). Analysis of Proline Metabolic Enzymes in Oryza Sativa under NaCl Stress. J. Environ. Biol..

[131] Huang Z., Zhao L., Chen D., Liang M., Liu Z., Shao H., Long X. (2013). Salt Stress Encourages Proline Accumulation by Regulating Proline Biosynthesis and Degradation in Jerusalem Artichoke Plantlets. PLoS ONE.

[132] Kausar F., Shahbaz M. (2013). Interactive Effect of Foliar Application of Nitric Oxide (NO) and Salinity on Wheat (Triticum Aestivum L.). Pak. J. Bot.

[133] Qu X., Wang H., Chen M., Liao J., Yuan J., Niu G. (2019). Drought Stress–Induced Physiological and Metabolic Changes in Leaves of Two Oil Tea Cultivars. J. Am. Soc. Hortic. Sci..

[134] Wang M., Liu C., Li S., Zhu D., Zhao Q., Yu J. (2013). Improved Nutritive Quality and Salt Resistance in Transgenic Maize by Simultaneously Overexpression of a Natural Lysine-Rich Protein Gene, SBgLR, and an ERF Transcription Factor Gene, TSRF1. Int. J. Mol. Sci..

[135] Jespersen D., Yu J., Huang B. (2017). Metabolic Effects of Acibenzolar-s-Methyl for Improving Heat or Drought Stress in Creeping Bentgrass. Front. Plant Sci..

[136] Kusvuran S., Dasgan H.Y., Abak K. (2013). Citrulline Is an Important Biochemical Indicator in Tolerance to Saline and Drought Stresses in Melon. Sci. World J..

[137] Joshi V., Joung J.-G., Fei Z., Jander G. (2010). Interdependence of Threonine, Methionine and Isoleucine Metabolism in Plants: Accumulation and Transcriptional Regulation under Abiotic Stress. Amino Acids.

[138] Gao S., Ouyang C., Wang S., Xu Y., Tang L., Chen F. (2008). Effects of Salt Stress on Growth, Antioxidant Enzyme and Phenylalanine Ammonia-Lyase Activities in *Jatropha curcas* L. Seedlings. Plant, Soil Environ..

[139] Haglund B.M. (1980). Proline and Valine—Cues Which Stimulate Grasshopper Herbivory during Drought Stress?. Nature.

[140] Khodary S.E.A. (1992). Effect of Salinity and Tryptophan on Growth and Some Metabolic Changes in Wheat and Sorghum Plants. Biol. Plant..

[141] Muthuramalingam P., Krishnan S.R., Pandian S., Mareeswaran N., Aruni W., Pandian S.K., Ramesh M. (2018). Global Analysis of Threonine Metabolism Genes Unravel Key Players in Rice to Improve the Abiotic Stress Tolerance. Sci. Rep..

[142] Wu D., Cai S., Chen M., Ye L., Chen Z., Zhang H., Dai F., Wu F., Zhang G. (2013). Tissue Metabolic Responses to Salt Stress in Wild and Cultivated Barley. PLoS ONE.

[143] Hussein M.M., Gaballah M.S., El-Faham S.Y. (2005). Amino Acids in Grains of Barley Plants as Affected by Benzyl Adenine and Salinity from Diluted Sea Water. J. Appl. Sci..

[144] Sacchi R., Li J., Villarreal F., Gardell A.M., Kültz D. (2013). Salinity-Induced Regulation of the Myo-Inositol Biosynthesis Pathway in Tilapia Gill Epithelium. J. Exp. Biol..

[145] Winter G., Todd C.D., Trovato M., Forlani G., Funck D. (2015). Physiological Implications of Arginine Metabolism in Plants. Front. Plant Sci..

[146] Zarei A., Brikis C.J., Bajwa V.S., Chiu G.Z., Simpson J.P., DeEll J.R., Bozzo G.G., Shelp B.J. (2017). Plant Glyoxylate/Succinic Semialdehyde Reductases: Comparative Biochemical Properties, Function during Chilling Stress, and Subcellular Localization. Front. Plant Sci..

[147] Page A.F., Minocha R., Minocha S.C. (2012). Living with High Putrescine: Expression of Ornithine and Arginine Biosynthetic Pathway Genes in High and Low Putrescine Producing Poplar Cells. Amino Acids.

[148] Li S., Peng F., Xiao Y., Gong Q., Bao Z., Li Y., Wu X. (2020). Mechanisms of High Concentration Valine-Mediated Inhibition of Peach Tree Shoot Growth. Front. Plant Sci..

[149] Bonner C.A., Rodrigues A.M., Miller J.A., Jensen R.A. (1992). Amino Acids Are General Growth Inhibitors of Nicotiana Silvestris in Tissue Culture. Physiol. Plant..

[150] Forsum O., Svennerstam H., Ganeteg U., Nsholm T. (2008). Capacities and Constraints of Amino Acid Utilization in Arabidopsis. New Phytol..

[151] Lea P.J., Sodek L., Parry M.A.J., Shewry P.R., Halford N.G. (2007). Asparagine in Plants. Ann. Appl. Biol..

[152] Hamberger B., Hahlbrock K. (2004). The 4-Coumarate:CoA Ligase Gene Family in Arabidopsis Thaliana Comprises One Rare, Sinapate-Activating and Three Commonly Occurring Isoenzymes. Proc. Natl. Acad. Sci. USA.

[153] Maeda H., Yoo H., Dudareva N. (2011). Prephenate Aminotransferase Directs Plant Phenylalanine Biosynthesis via Arogenate. Nat. Chem. Biol..

[154] Allan W.L., Clark S.M., Hoover G.J., Shelp B.J. (2009). Role of Plant Glyoxylate Reductases during Stress: A Hypothesis. Biochem. J..

[155] Kiani-Pouya A., Roessner U., Jayasinghe N.S., Lutz A., Rupasinghe T., Bazihizina N., Bohm J., Alharbi S., Hedrich R., Shabala S. (2017). Epidermal Bladder Cells Confer Salinity Stress Tolerance in the Halophyte Quinoa and Atriplex Species. Plant. Cell Environ..

[156] Kumari A., Parida A.K. (2018). Metabolomics and Network Analysis Reveal the Potential Metabolites and Biological Pathways Involved in Salinity Tolerance of the Halophyte *Salvadora persica*. Environ. Exp. Bot..

[157] Liu X., Wu H., Ji C., Wei L., Zhao J., Yu J. (2013). An Integrated Proteomic and Metabolomic Study on the Chronic Effects of Mercury in *Suaeda salsa* under an Environmentally Relevant Salinity. PLoS ONE.

[158] Dong S., Zhang J., Beckles D.M. (2018). A Pivotal Role for Starch in the Reconfiguration of 14C-Partitioning and Allocation in *Arabidopsis thaliana* under Short-Term Abiotic Stress. Sci. Rep..

[159] Variyar P.S., Banerjee A., Akkarakaran J.J., Suprasanna P. (2014). Role of Glucosinolates in Plant Stress Tolerance. Emerging Technologies and Management of Crop Stress Tolerance.

[160] Michaletti A., Naghavi M.R., Toorchi M., Zolla L., Rinalducci S. (2018). Metabolomics and Proteomics Reveal Drought-Stress Responses of Leaf Tissues from Spring-Wheat. Sci. Rep..

