# Supplementary material for: Physiological and Metabolic Responses of Gac Leaf (Momordica cochinchinensis (Lour.) Spreng.) to Salinity Stress
Source: Plants (Basel). 2022 Sep 20;11(19):2447. doi: 10.3390/plants11192447 (PMC9572180; doi:10.3390/plants11192447)
Supplement: Supplementary file 1 [file plants-11-02447-s001.zip › plants-1896763-supplementary-Figures.pdf]

Supplementary Material

Physiological and Metabolic responses of Gac Leaf (*Momordica cochinchinensis* (Lour.) Spreng.) to Salinity Stress

Thitiwan Jumpa [1], Diane M. Beckles [2,*], Patcharin Songsri [3], Kunlaya Pattanagul [4] and Wattana Pattanagul [1,*]

1 Department of Biology, Faculty of Science, Khon Kaen University, Khon Kaen 40002, Thailand; thitiwan_j@kkumail.com

2 Department of Plant Sciences, University of California, Davis, CA 95615, USA

3 Department of Plant Sciences and Agricultural Resources, Faculty of Agriculture, Khon Kaen University, Khon Kaen 40002, Thailand; patcharinso@kku.ac.th

4 Department of Statistics, Faculty of Science, Khon Kaen University, Khon Kaen 40002, Thailand; kunlaya_17@kku.ac.th

* Correspondence: dmbeckles@ucdavis.edu (D.M.B.); wattana@biology.in.th (W.P.)

List of supplementary Figures

Figure S1. Soil electrical conductivity

Figure S2. Scatter plot of electrolyte leakage vs. soil EC

Figure S3. Assessment of chlorophyll by SPAD in leaves of salinity-treated gac seedlings

Figure S4. PCA score plots

Figure S5. PCA scree plots

Figure S6. Pathway impact metabolic changes compared to control found in 100 mM

Figure S7. Pathway impact metabolic changes compared to control found in 150 mM

Figure S8. Volcano plots of significant metabolites in each treatment compared to control

Figure S9. One-way ANOVA analysis

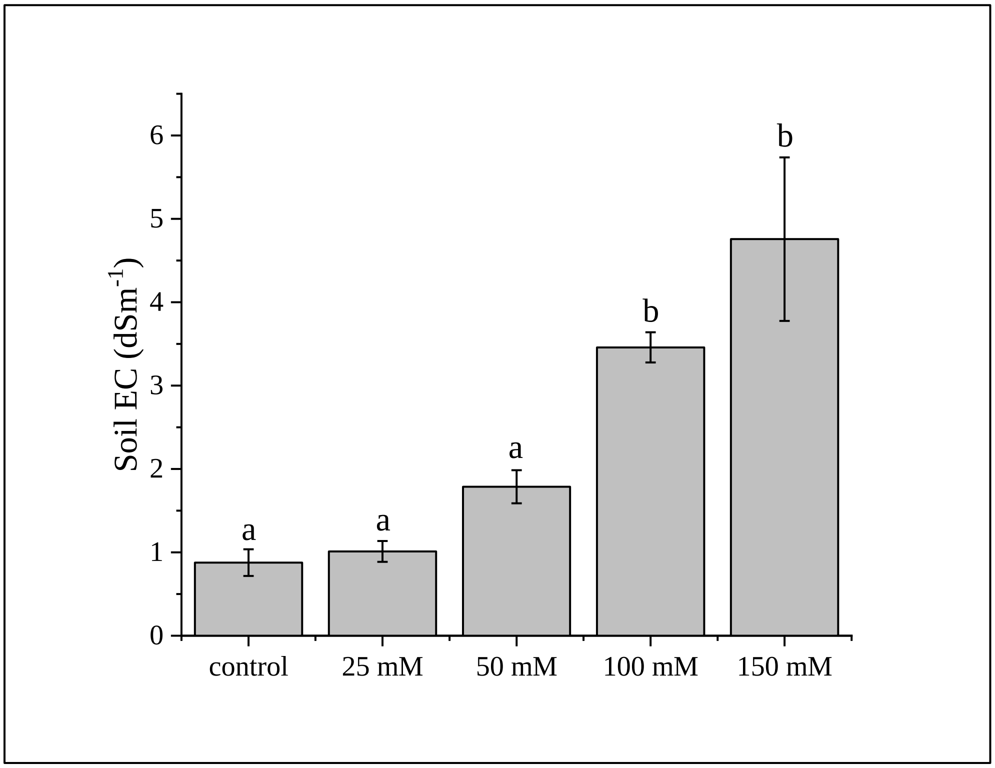

Figure S1. Soil electrical conductivity (soil EC) levels when increasing soil salinity. Values are means ± SE (n=6). Different lowercase letters indicate significant differences between treatments ($p < 0.05$).

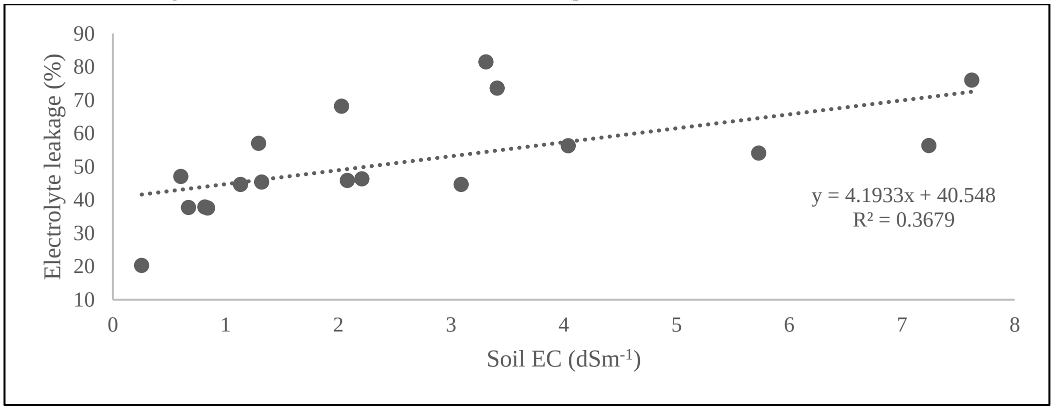

Figure S2. A scatter plot of electrolyte leakage vs. soil EC.

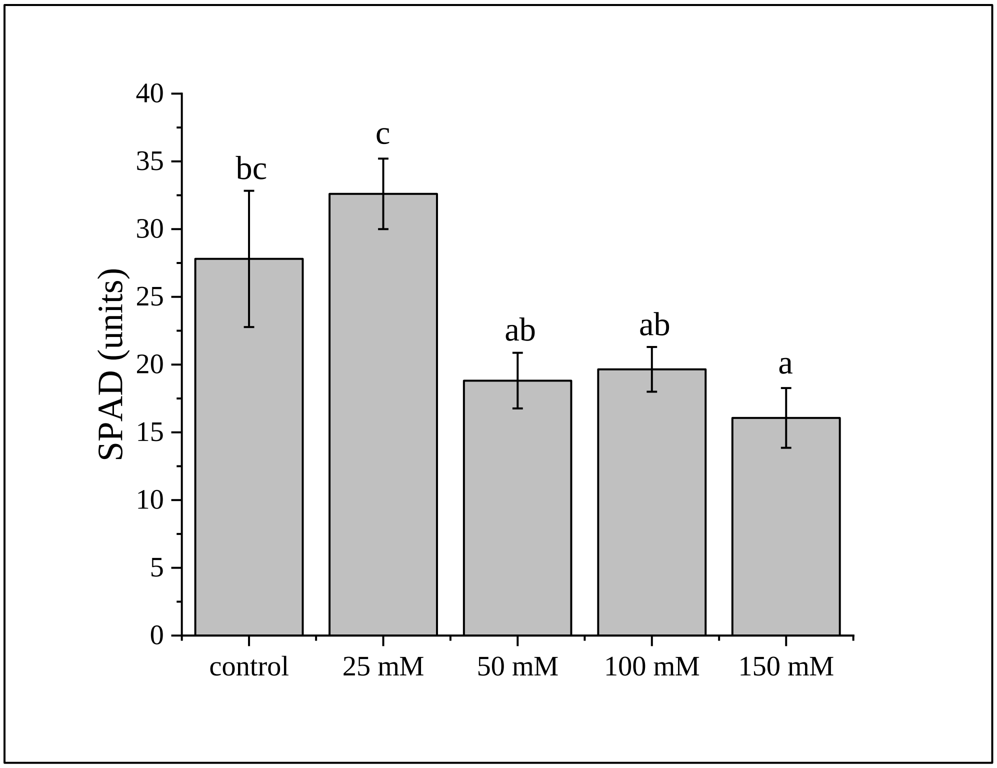

Figure S3. Assessment of chlorophyll by SPAD in leaves of salinity-treated gac seedlings. (**A**) Chlorophyll *a*, *b* and total chlorophyll and (**B**) chlorophyll assessment by SPAD. Values are means ± SE (n=6). Different lowercase letters indicate significant differences between treatments ($p < 0.05$).

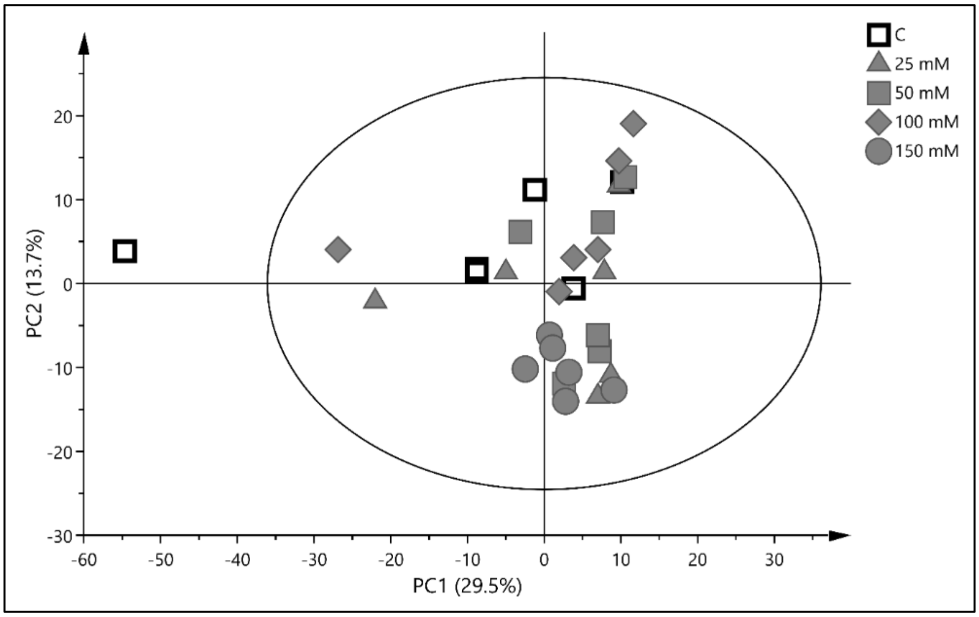

Figure S4. **PCA score plot** of metabolites measured by GC-MS in gac leaf grown under different concentrations of salinity stress including 0, 25, 50, 100, and 150 mM NaCl. Each of six biological replicates was individually plotted and the samples were projected onto a bi-plot showing the first two PCs. Each symbol on the plot represents data from 637 metabolites reduced to a single data point defined by the first (PC1) and second (PC2) PC. Samples that have similar metabolite composition will cluster together while samples that are different will be further apart.

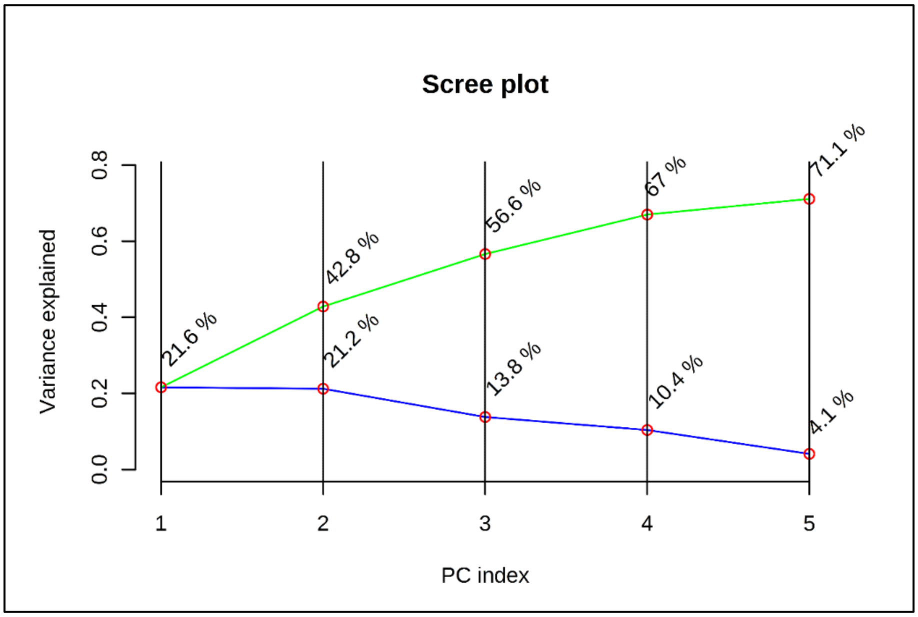

Figure S5. PCA scree plot of metabolites measured by GC-MS in gac leaf grown under different concentrations of salinity stress including 0, 25, 50, 100, and 150 mM NaCl.

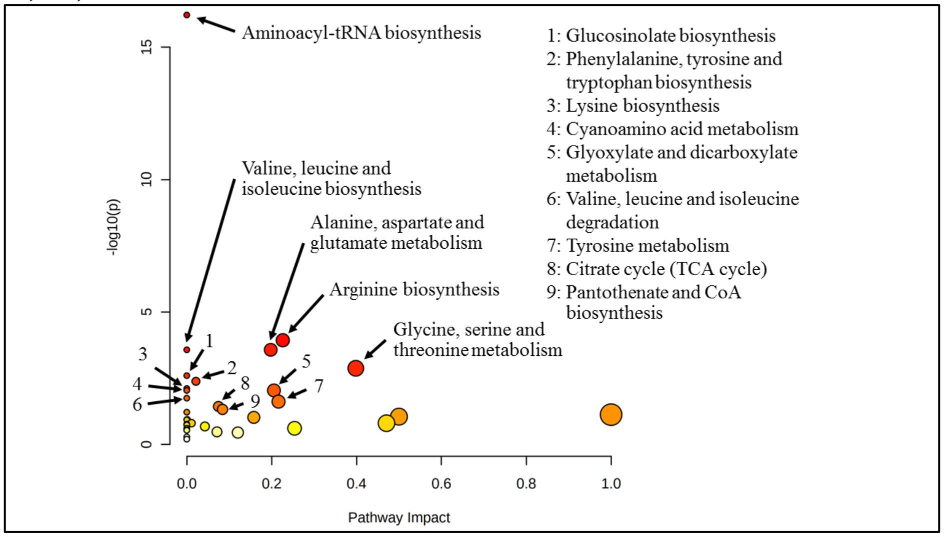

Figure S6. Pathway impact metabolic changes compared to control found in 100 mM. The metabolome view shows all matched pathways according to the *p* values from the pathway enrichment analysis and pathway impact values from the pathway topology analysis. The colors (varying from yellow to red) means the metabolites are in the data with different levels of significance.

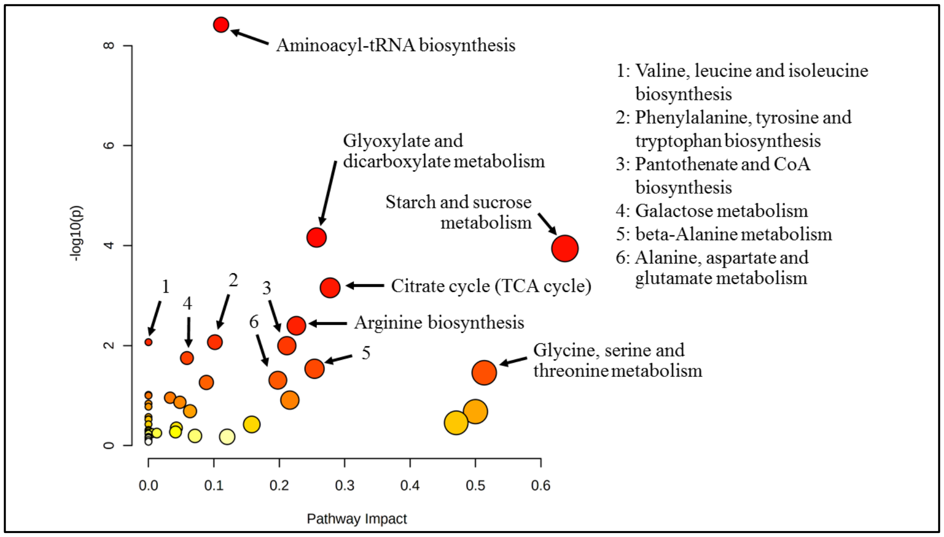

Figure S7. Pathway impact metabolic changes compared to control found in 150 mM. The metabolome view shows all matched pathways according to the *p* values from the pathway enrichment analysis and pathway impact values from the pathway topology analysis. The colors (varying from yellow to red) means the metabolites are in the data with different levels of significance.

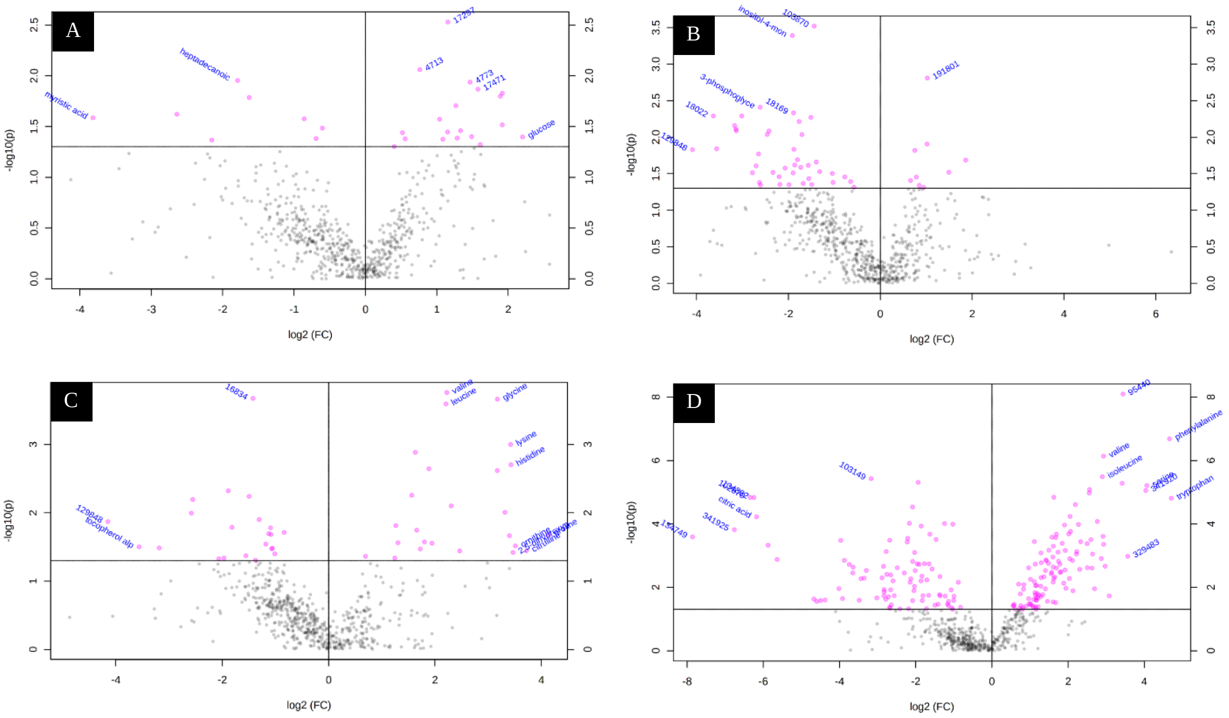

Figure S8. **Volcano plots of significant metabolites in each treatment compared to control**. The signal detection result shows the magnitude (log2 fold change (FC), x-axis) and significance (−log10 adjusted *p*-value, y-axis). Each pink spot represents a specific metabolites-treatment interaction with a statistical significance threshold ($P \leq 0.05$ after adjustment with Bonferroni correction) and the threshold of $FC > 1$ or < -1. The spots on the left represent the metabolites more frequently associated with control; the spots on the right, metabolites more frequently associated with (**A**) 25 mM, (**B**) 50 mM, (**C**) 100 mM and (**D**) 150 mM. Volcano plot was generated using Metaboanalyst 5.0 (https://www.metaboanalyst.ca/).

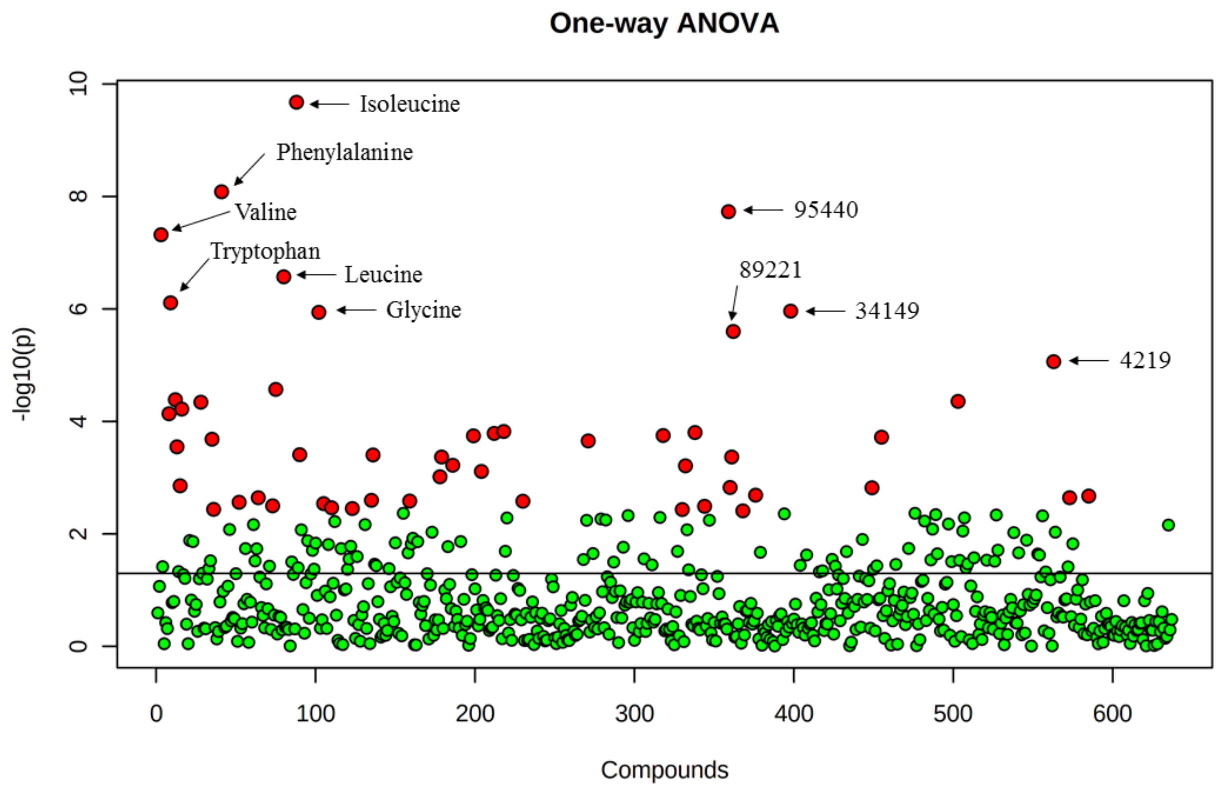

Figure S9. One-way ANOVA analysis of metabolites measured by GC-MS-TOF in gac leaf grown under 0, 25, 50, 100, and 150 mM NaCl. Each metabolite was individually plotted at the adjusted *p* value = 0.05, Posthoc analysis: Fisher's LSD. The red dots represented significant metabolites (52 dots) and the green dots represented non-significant metabolites (585 dots).
